# Hurricane-Driven Patterns of Clonality in an Ecosystem Engineer: The Caribbean Coral *Montastraea annularis*

**DOI:** 10.1371/journal.pone.0053283

**Published:** 2013-01-07

**Authors:** Nicola L. Foster, Iliana B. Baums, Juan A. Sanchez, Claire B. Paris, Iliana Chollett, Claudia L. Agudelo, Mark J. A. Vermeij, Peter J. Mumby

**Affiliations:** 1 Marine Spatial Ecology Lab, School of BioSciences, University of Exeter, Exeter, United Kingdom; 2 Department of Biology, Pennsylvania State University, University Park, Pennsylvania, United States of America; 3 Laboratorio de Biología Molecular Marina (BIOMMAR), Departamento Ciencias Biológicas-Facultad de Ciencias, Universidad de los Andes, Bogotá, Colombia; 4 Rosenstiel School of Marine & Atmospheric Science, University of Miami, Miami, Florida, United States of America; 5 Carmabi Foundation, Willemstad, Curaçao; 6 Aquatic Microbiology, Institute for Biodiversity and Ecosystem Dynamics (IBED), University of Amsterdam, Amsterdam, The Netherlands; King Abdullah University of Science and Technology, Saudi Arabia

## Abstract

K-selected species with low rates of sexual recruitment may utilise storage effects where low adult mortality allows a number of individuals to persist through time until a favourable recruitment period occurs. Alternative methods of recruitment may become increasingly important for such species if the availability of favourable conditions for sexual recruitment decline under rising anthropogenic disturbance and climate change. Here, we test the hypotheses that asexual dispersal is an integral life history strategy not only in branching corals, as previously reported, but also in a columnar, ‘K-selected’ coral species, and that its prevalence is driven by the frequency of severe hurricane disturbance. Montastraea annularis is a long-lived major frame-work builder of Caribbean coral reefs but its survival is threatened by the consequences of climate induced disturbance, such as bleaching, ocean acidification and increased prevalence of disease. 700 M. annularis samples from 18 reefs within the Caribbean were genotyped using six polymorphic microsatellite loci. We demonstrate that asexual reproduction occurs at varying frequency across the species-range and significantly contributes to the local abundance of *M.* annularis, with its contribution increasing in areas with greater hurricane frequency. We tested several competing hypotheses that might explain the observed pattern of genotypic diversity. 64% of the variation in genotypic diversity among the sites was explained by hurricane incidence and reef slope, demonstrating that large-scale disturbances combine with local habitat characteristics to shape the balance between sexual and asexual reproduction in populations of M. annularis.

## Introduction

Sessile, clonal organisms are an important component of terrestrial and aquatic communities [1]–[5]. They grow through replication of modules (budding of polyps or vegetative propagation) and can reproduce asexually using a variety of mechanisms including mechanical fragmentation of individuals, fission through partial mortality, and release of asexually generated propagules [6]–[9]. Sessile, clonal organisms often also produce sexually generated offspring [3], [6], which typically increases genetic diversity. Sexually and asexually derived propagules have the ability to disperse beyond the natal population, whereas the dispersal ability of offspring generated by fragmentation or fission is much more limited [10]. The proportions of sexual and asexual recruitment within populations of clonal organisms can be influenced by biotic and abiotic factors [11]–[15]. Disturbance in particular has been highlighted as a mechanism that enhances and retards asexual recruitment in populations of aquatic [16]–[18] and terrestrial species [10], [19], [20].

The ability of a species to reproduce sexually and asexually is suggested to influence ecological and evolutionary processes such as local adaptation, space pre-emption, species longevity and gene flow [6], [10], [21], [22]. While sexually produced propagules may establish widely dispersed, genetically diverse populations, asexual recruitment via fragmentation or fission may assist in the rapid expansion of a population within an area and facilitate the adaptation of a few well-suited genotypes to local ecological conditions [15], [23], [24], or aid in the colonisation of habitats unsuitable for propagule settlement [25]. Furthermore, asexual reproduction can increase the longevity of a species beyond that of an ecologically equivalent aclonal species [26], spread the risk of mortality among individuals within a genet and increase cumulative fecundity [9], [25].

Clonal organisms exhibit a variety of life history strategies in which the relative importance of sexual and asexual reproduction varies. At one extreme are classic ‘*r*-selected’ species. These are typically fast growing and short lived species with a low competitive ability, therefore their survival is dependent on the capacity of the population to produce large numbers of offspring via sexual or asexual reproduction [27], [28]. At the other extreme are ‘*K*-selected’ organisms. These species are typically slow growing, characterised by high longevity, are well adapted to their environment and are successful competitors, which enables populations to dominate and expand to the carrying capacity of the habitat [27], [28]. The ability of *K*-strategists to dominate in the presence of low rates of sexual reproduction has led to the hypothesis that some species utilise storage effects, whereby the persistence of a small group of adults maintains the population when recruitment fails [29]. Low adult mortality within these organisms allows a number of individuals to persist through time until a favourable recruitment period occurs [29]. However, potential problems may exist for species employing storage effects if favourable conditions for recruitment occur so infrequently that they fall outside the life span of the cohort. Here, we study a system in which the capacity of storage effects may have been severely compromised in recent years and is predicted to deteriorate further.

Coral reefs have experienced large shifts in community structure in recent years, with many reefs, particularly in the Caribbean, undergoing a transition from coral-dominated to algal-dominated reefs [30]–[32]. Declining reef health has been attributed to natural and anthropogenic disturbances including: a reduction in key herbivorous species through overfishing and disease [33], [34], increased frequency and severity of hurricanes [35]–[37], increased frequency of mass-bleaching events [38] and increased frequency and prevalence of disease [39]. The rapid proliferation of macroalgae observed on reefs can reduce the rate of coral recruitment [40], [41]. Therefore, favourable conditions for the recruitment of sexually generated larvae are likely to have declined in recent decades and may become even less frequent [42]. Thus, further inhibiting the recruitment success of reef species, including those that utilise storage effects. Moreover, the frequency of favourable conditions for sexual recruitment may decline beyond that necessary to sustain population levels.

The massive coral Montastraea annularis (Ellis and Solander), sensu stricto, is a dominant frame-work builder of Caribbean coral reefs, forming dome-shaped colonies frequently over 1 m in diameter, and composed of columns. The abundance of this important coral species has declined in the past 25 years with some populations in St John, US Virgin Islands, showing a 30% decrease in cover over an eleven year period [43]. *M. annularis* colonies are characterised by a slow growth rate of <10 mm yr^−1^ [44], [45] and a high longevity, with many colonies in a population estimated to be more than 100 years old. Sexual reproduction occurs annually utilising a mass-spawning event [46] yet the recruitment rate of sexually generated larvae into adult populations is low [43], [47], [48]. Such a paucity of sexual recruits in spite of annual broadcast spawning of gametes, high colony fecundity and relatively high fertilisation rates [46], [49], [50], suggests that populations of *M. annularis* may utilise a storage effect, where significant recruitment of sexually derived larvae only occurs on the scale of decades when conditions are favourable [41]. Using a size-based demographic model, Edmunds & Elahi [43] demonstrated that episodic recruitment on a scale of once every 25 years was unable to sustain current population levels of *M. annularis* at St. John, U.S. Virgin Islands. Therefore, alternative modes of colony dispersal, such as asexual reproduction, are likely to become increasingly important for the persistence of M. annularis.

Although massive, ‘*K*-selected’ corals grow through asexual budding (like all scleractinians), the widespread existence of asexual methods of colony dispersal has only recently been discovered [51], [52]. In Honduras, spatially-discrete colonies of *M. annularis* were, on occasion, clonemates [51]. Because the level of clonality was highest at the site with greatest physical disturbance (explained at this local scale by differences in wave exposure), we hypothesized that physical colony breakage was the most likely mechanism generating clones. Here, we investigate the generality of this observation across the Caribbean and test a refined hypothesis: asexual dispersal is an integral life history strategy of M. annularis and its prevalence is driven by physical disturbance, which at this basin-scale is mainly related to changes in the occurrence of hurricanes. Because hurricane incidence varies by at least an order of magnitude across the Caribbean region [53], the frequency of clonemates should reflect such large-scale geographic patterns. In addition, we test several other competing hypotheses that may help to explain the observed patterns of clonality among populations of *M. annularis*, including exposure, colony size, reef slope and larval supply.

## Methods

We refer to a group of genetically identical colonies descended from a single zygote as a “genet” [54] and term spatially independent colonies within the genet “clonemates”. Spatially independent colonies are defined as colonies with no interconnecting tissue or skeleton.

### Sampling

The necessary permits for collection and export of coral samples were provided by the Department of Fisheries, Nassau, The Bahamas; Fisheries Department, Belize City, Belize; Secretaria de Agricultura y Ganaderia Despacho Ministerial, Tegucigalpa, Honduras; Department of Public Health, Willemstad, Curaçao; Government of Colombia. CITES import permits were provided by the Department for the Environment, Food and Rural Affairs, Bristol, UK.

**Figure 1:**
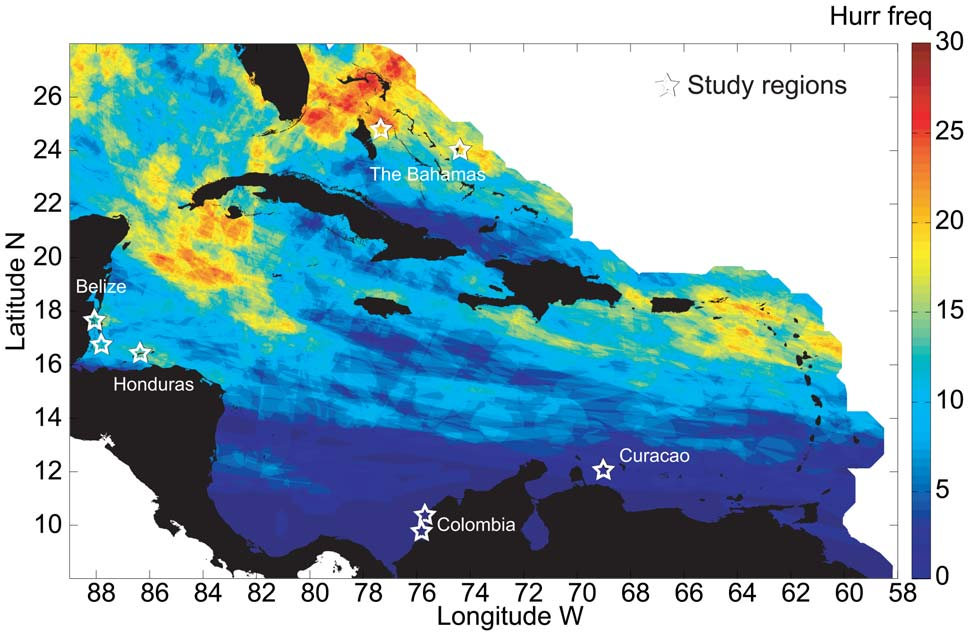
Study countries and regions in the Caribbean from which samples of Montastraea annularis were collected. Base map indicates the frequency of occurrence of hurricanes category 1–5 in the last 141 years (1863–2004; [53]).

Tissue samples were collected from a total of 700 *Montastraea annularis (sensu stricto)* colonies at 18 reefs in 8 regions of the Caribbean (Fig. 1; Table 1). The Caribbean basin was divided into three latitudinal bands (high, medium and low hurricane frequency) using the average number of hurricanes to strike an area in any given year (Fig. 1) [55]. Hurricanes have impacted the Caribbean in a spatially heterogeneous way. Although average hurricane incidence for the period 1863–2004 is 8.86±6.0 for the entire Caribbean basin, some areas have not been affected by hurricanes at all and others have been impacted 32 times. Using this information, we selected locations that have been impacted by low (0–3 hurricanes, Curacao and Colombia), medium (10–15, Belize and Honduras) and high (20–25, the Bahamas) hurricane frequency (Fig. 1). Within each band, locations were selected based on the presence of *M. annularis* reefs and the feasibility of sampling. In each of the locations, two or three reefs were selected a minimum of 2 km apart.

**Table 1:** Location, reef, approximate GPS coordinates and hurricane frequency for 18 *Montastraea annularis* sites sampled in the Caribbean.

Country	Region	Reef^a^	Latitude (°)	Longitude (°)	Hurricane Frequency
Bahamas	New Providence	School House Reef (SCR)	24.588	−77.3005	High
		Propeller Reef (PR)	25.0046	−77.3309	High
	San Salvador	Seahorse Reef (SHR)	24.0878	−74.2852	High
		Snapshot Reef (SSR)	24.0230	−74.3158	High
Belize	Glovers Reef	Long Cay (LC)	16.7540	−87.7814	Medium
		West Reef (WR)	16.7582	−87.8768	Medium
	Caye Caulker	Coral Gardens (CG)	17.7484	−88.0233	Medium
		Eagle Ray (ER)	17.7203	−88.0136	Medium
Colombia	Cartagena	Palo (PA)	10.268	−75.622	Low
		Palo 1 (PA1)	10.277	−75.611	Low
		Pendales (PE)	10.238	−75.597	Low
	San Bernardo	Rosario Sur (RS)	10.161	−75.788	Low
		San Bernardo (SBE)	9.777	−75.908	Low
Curaçao	Curaçao	Buoy 1 (BU1)	12.1259	−69.0523	Low
		Snakebay (SNB)	12.139	−69.0021	Low
Honduras	Roatan	Seaquest (SQ)	16.294	−86.600	Medium
		Sandy Bay (SB)	16.334	−86.568	Medium
		Western Wall (WW)	16.271	−86.604	Medium

a Reef abbreviation used in text and figures is provided in parentheses.

Each site was located on the forereef at a depth of between 3–7 m and a circular sampling plot was established with a minimum area of 78.5 m^2^. Every *M. annularis* colony within each plot was sampled and its location recorded by noting the distance (to nearest 5 cm) and bearing (to nearest 5 degrees) from the centre of the sampling plot. Due to the low density of colonies at three sites in Colombia (Palo, Palo 1 and San Bernardo) the circular sampling plot was replaced by a haphazard transect across the reef (the area covered by the transect was not measured). Individual colonies were sampled as they were located along the transect and the distance and bearing to the previous colony was recorded (in the majority of cases). Where colony size was measured, the length, width and height of the colony to the nearest 5 cm were recorded. Colony condition was estimated as percent of live tissue. One sample (1 cm×1 cm) was taken from the edge of a lobe on each colony using a hammer and chisel and placed in a labelled zip lock bag. On returning to shore each sample was preserved in 70% alcohol and stored at 4°C prior to DNA extraction.

### Genotyping

Six polymorphic microsatellite loci, developed by Severance *et al* [56], were used to identify genets within populations of *M. annularis.* DNA extraction and genotyping of samples were undertaken as described in Foster *et al* [51]. In brief, two multiplex polymerase chain reactions (PCR) were carried out per sample using fluorescently labelled primers. PCR products were visualized using a CEQ 8000 (Beckman Coulter) automated DNA sequencer with an internal size standard (Size Standard 400) for accurate sizing. For samples collected in Colombia, PCR products were visualised using an ABI 310 (Applied Biosystems) automated DNA sequencer with an internal size standard (Gene Scan 500-LIZ) for accurate sizing. Electropherograms were analysed using GeneMarker Software 1.5 (Soft Genetics) and alleles were scored based on amplicon size. Owing to the presence of null alleles in certain populations, samples from Honduras and Colombia were analysed using only four of the six microsatellite loci and samples from Glovers Reef were analysed using only five of the six microsatellite loci. A selection of random samples was re-amplified (n = 34) and allele scores were consistent with the first amplification with minimal error (inconsistent allele scored in 8 out of 312 cases). Genotyping results for Honduras (generated in the same laboratory with the same equipment) were taken from Foster *et al* [51].

## Analyses

### Sampling

Unless a population is completely dominated by a single genet, sampling effort can affect the number of genets (N_g_) detected within a population. A minimum target size of 35 colonies sampled per location was established *a priori*. We started with a sampling area of 78.5 m^2^ and expanded this area only if the target sampling size could not be reached. Thus, sampling effort was kept constant and the results from different reefs were directly comparable.

### Genotyping

Of the 700 samples collected, 698 were successfully genotyped. Samples which had identical alleles at all analysed loci were identified as clonemates belonging to the same genet. Identical multilocus genotypes were never shared between sites, only within sites. The probability of identity (*P*_ID_) was calculated to provide a conservative estimate of the probability that two colonies sampled from the same site share a multilocus genotype by chance, not by descent [57]. Biased and unbiased *P*_ID_ was calculated for each locus by GIMLET [58] and multiplied across loci to give the combined *P*_ID_ for each site [57]. The small *P_ID_* values calculated for the sites (Table 2) indicate the low probability of misidentifying colonies as clonemates when they are not. Microchecker was used to check for the presence of null alleles [59].

**Table 2:** Probability of identity (*P_ID_*) for each locus within each region for *Montastraea annularis* sampled across the Caribbean.

Region	Locus	Biased *P_ID_*	Unbiased *P_ID_*	Combined Biased *P_ID_*	Combined Unbiased *P_ID_*
San Salvador	5	0.03996	0.03223	7×10^−10^	1.1×10^−10^
	11	0.00563	0.00268		
	12	0.00749	0.00403		
	28	0.083	0.07209		
	4	0.06829	0.05952		
	8	0.7304	0.7195		
New Providence	5	0.06064	0.05219	4.2×10^−9^	2.8×10^−10^
	11	0.01166	0.00752		
	12	0.00483	0.00238		
	28	0.08446	0.07448		
	4	0.08996	0.0815		
	8	0.8844	0.88		
Caye Caulker	5	0.04911	0.03375	3.3×10^−10^	7.6×10^−12^
	11	0.00782	0.00252		
	12	0.00525	0.00108		
	28	0.04569	0.03385		
	4	0.03979	0.02756		
	8	0.8936	0.8857		
Glovers Reef	5	0.02781	0.02292	2.5×10^−8^	8.9×10^−9^
	12	0.00509	0.00290		
	28	0.04114	0.03555		
	4	0.04965	0.04325		
	8	0.8769	0.8729		
Curaçao	5	0.05122	0.04091	7.7×10^−10^	6.3×10^−11^
	11	0.00835	0.00369		
	12	0.00599	0.00208		
	28	0.07769	0.06574		
	4	0.06122	0.04971		
	8	0.6319	0.6133		
Roatan	5	0.02682	0.02365	8.7×10^−6^	6.6×10^−6^
	28	0.0744	0.06957		
	4	0.06225	0.05738		
	8	0.7033	0.6975		
Cartagena	5	0.02967	0.0291	1.2×10^−6^	5.6×10^−7^
	28	0.04048	0.03269		
	4	0.01285	0.008035		
	8	0.7462	0.7354		
San Bernardo	5	0.0541	0.03841	3.0×10^−6^	4.5×10^−7^
	28	0.04227	0.02699		
	4	0.01303	0.004379		
	8	1	1		

Note: combined probability of identity for each region also provided.

### Genotypic Diversity

Genotypic evenness was calculated as G_o_/N_g_ [60] where G_o_ is the observed genotypic diversity. G_o_ was calculated as:
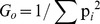
where *p_i_* is the frequency of the *i*th genotype in the population [61]. Genotypic evenness equals zero in a population dominated by a single genet and one where each genet is represented by an equal number of individuals. The contribution of sexual and asexual reproduction to population structure (genotypic diversity) was estimated by calculating the ratio of the observed genotypic diversity, G_o,_ over the expected genotypic diversity, G_e_ [61]. Genotypic diversity (G_o_/G_e_) equals one in a solely sexual population and zero in a population dominated by a single genet. Based on the combination of genotypic diversity and evenness, populations were classified into three groups (sexual, mostly sexual and mostly asexual) to facilitate analysis [15].

### Colony Size

The size of colonies (volume, m^3^) within each site was calculated as the product of colony length, width and height (Table 3). As the size distribution of colonies was skewed towards smaller sizes the data were log transformed. We predicted that the size of colonies within a site would be influenced by hurricane incidence, with those sites that had experienced a greater number of hurricanes having smaller average colony size. Log mean colony size within each site was analysed across sites and entered into a linear regression with hurricane incidence as the independent variable and a linear mixed effects model with genotypic diversity as the dependent variable. Log mean colony size was compared between clonemates and non-clonemates and among the three clonal structure groups identified.

**Table 3:** Physical parameters for 18 populations of *Montastraea annularis* across the Caribbean.

Region	Reef^a^	Hurricane Incidence^b^	Reef Slope (°)	Exposure Index^c^	Log Colony Size^d^ (m^3^)	Larval Input (Number of particles)
Roatan	SB	8	3.19	0.039	−0.973±0.08	192.2
	SQ	8	1.79	2.05×10^−6^	−0.686±0.07	192.2
	WW	8	7.75	2.44×10^−4^	−0.753±0.11	385
Caye Caulker	CG	16	0.50	0.107	−0.788±0.11	545.6
	ER	16	0.79	0.125	−0.572±0.14	814
Glovers Reef	LC	6	4.48	1.9×10^−7^	−0.295±0.10	189.2
	WR	6	5.05	0.002	−0.458±0.07	620.8
San Salvador	SHR	17	0.83	2.150	−0.651±0.10	280
	SSR	17	1.55	0.493	−0.924±0.10	360
New Providence	SCR	19	1.06	3.127	−1.438±0.13	1393.2
	PR	19	1.29	4.901	−1.114±0.08	2.2
Curaçao	BU1	3	8.07	1.71×10^−16^	−1.506±0.11	64.4
	SNB	3	4.94	5.31×10^−23^	−1.499±0.11	64.4
Cartagena	PA	0	0.08	0.035	0.415±0.10	35.2
	PA1	0	0.08	0.009	0.168±0.09	35.2
	PE	0	0.11	2.80×10^−8^	−0.203±0.23	35.2
San Bernardo	RS	0	2.00	1.21×10^−6^	−0.575±0.36	236
	SBE	0	1.15	4.918	0.394±0.15	370.4

a Reef abbreviations provided in Table 1.

b Number of hurricanes to pass a location between 1863 and 2004.

c Exposure values of 10^−23^ are effectively zero.

d Mean±SE.

### Spatial Distribution of Clonemates

The spatial distribution of colonies at each site was mapped on to polar plots using the radial sampling coordinates. *XY* distances were then calculated for each colony and the pairwise distances between clonemates were calculated. To discriminate the mechanism by which potential clonemates arose, we assumed that storm-induced colony fragmentation must have occurred when the separation of clonemates exceeded that of the average adult colony size. If the distance between two clonemates was less than the width of an average adult colony (66 cm wide ±1.38 cm; based on the average width of colonies in the 18 sites) it was not possible to discount origins of partial-colony mortality (though severe colony erosion to the colony base only occurs rarely, Mumby pers. obs.).

### Hurricane Incidence

We predicted that a site with a high hurricane incidence was likely to have more asexually derived colonies than a site with a low hurricane incidence. Hurricane incidence was calculated by quantifying the number of storms experienced by each reef between 1863 and 2004. Hurricane-force winds may extend several kilometres from the hurricane track. We calculated the frequency of hurricanes at any given reef site using a standard protocol, where the area of influence of each hurricane is captured by a buffer of varying width according to the intensity of the storm [15], [55]: a 35 km buffer zone for Tropical storms (TS) and category 1 and 2 hurricanes (HS1 and HS2), a 60 km buffer zone for category 3 hurricanes (HS3) and a 100 km buffer zone for category 4 and 5 hurricanes (HS4 and HS5). Storm tracks (http://maps.csc.noaa.gov/hurricanes) were queried for each reef using ArcGIS 9.1 and each storm was counted once when it entered its strength-specific buffer zone (Table 3). Hurricane incidence was entered into a linear mixed effects model with genotypic diversity as the dependent variable.

### Reef Slope

The gradient of the continental shelf on which a reef is located could affect the amount of asexual recruitment within populations, as observed by Baums et al [15]. Colonies within a population located on a steeper slope may be more susceptible to asexual reproduction as the slope could be exposed to higher disturbance strength. In addition, fragments might be moved farther from the parent colony in these conditions, whereas fragments are likely to be retained close to the parent colony on gently sloping shelves. Furthermore, a steeper slope may intensify the impacts of hurricanes through increased wave amplitude and power thereby causing more fragmentation [62], [63].

Reef slope was calculated using Erdas Imagine 8. Landsat TM images of each location were used to determine the reflectance of band 1 in three areas of pixels at the approximate location of each site (known depth) and two areas of pixels at approximately 20 m. Depth was plotted against the natural log of reflectance and the equation of the trendline could then be used to calculate depth at any point using the reflectance value of band 1. A transect line was drawn on the Landsat image from the site to approximately 20 m and the length in metres was recorded. The depth at the start and end of the transect was calculated using the equation generated above and change in depth was determined. The change in depth and length of transect were entered into the following equation to determine slope:
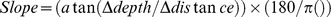



Reef slope (Table 3) was entered into a linear mixed effects model with genotypic diversity as the dependent variable.

### Exposure

Site exposure may influence the proportion of asexual recruitment within a population. Colonies within a site exposed to the prevailing winds may fragment more frequently than colonies within a site rarely exposed to strong winds and large waves. The level of exposure experienced by individual sites can be estimated using fetch and wind speed to calculate wave power. Wind speed data was obtained for every site over a twelve month period, between 2004 and 2007, from Weather Underground (www.weatherunderground.com). For each site, the closest weather station was selected and wind speed (m s^−1^) and direction were collected using historical data sets. Exposure was calculated using the protocol described by Harborne *et al* [64]. An index of exposure for each site (Table 3) was entered into a linear mixed effects model with genotypic diversity as the dependent variable.

### Larval Input

Larval input may influence the proportion of asexual recruitment within a population. Populations with higher fluxes of non-clonal corals (i.e. higher sexual recruitment) may have a lower proportion of asexually derived colonies purely because the input of larvae to the site is high. In order to test this alternative hypothesis, a larval connectivity model (described below) was used to estimate the amount of larval input (sexual recruitment) at each site. Larval input was then entered into a linear mixed effects model with genotypic diversity as the dependent variable.

The larval connectivity model was composed of four essential components that were adapted to *M. annularis* as follows: (1) the benthic seascape module used UNEP-WCMC [65] and Coral Reef Millennium Mapping Project [66] remote sensing data to generate n = 1,900 (ca. 5 km×10 km) coral reef polygons representing discrete spawning and settling habitats in the Caribbean; (2) the oceanographic module used the eddy-resolving, basin-scale Hybrid Coordinate Ocean Model (HYCOM 1/12°) with Global Ocean Data Assimilation Experiment (GODAE) providing daily 3-dimensional velocity predictions from 2004 to 2008 [67]; (3) the biological module parameterized for the spawning strategy and early life history traits of *M. annularis* (including mortality - see Table 4) prescribed passive advection of planulae during the pre-competency period and active settlement throughout competency following Baums *et al* [68]; lastly, (4) a Lagrangian stochastic module moved individual particles by integrating information from other modules at each time step (ΔT = 2 h). The larval connectivity model (detailed algorithm in Paris *et al* [69]) recorded the source, destination, and fate of each simulated planula spawned in each reef polygon during each reproductive cycle (Table 4), generating a matrix of larval migration **M**.

**Table 4:** Biological parameters of the Bio-oceanographic larval connectivity model for *Montastraea annularis.*

Spawning Mode			Broadcast	
**Time to Competency (days)**			1–6 d	
**Maximum PLD** a **(days)**			30 d	
**Larval half-life** b **(seconds)**			1296000	
**Spawning schedule & production**				
**Year**	**Month** c	**Days after Full Moon**		***N*** d
2004	9, 10	5		50
2005	8, 9	6		100
2006	8, 9	7		200
2007	9, 10	8		100
2008	8, 9	9		50

a PLD denotes Pelagic Larval Duration.

b Larval mortality was calculated using half-life.

c Months are numerical. Spawning months were determined by the calendar dates of full moons in late summer/early fall, and two spawning events were simulated each year. Spawning was simulated over five days (5–9 days after full moon), with peak spawning 7 days after the full moon.

d Number of larvae released per reef polygon.

## Results

### Genotypic Diversity

**Figure 2:**
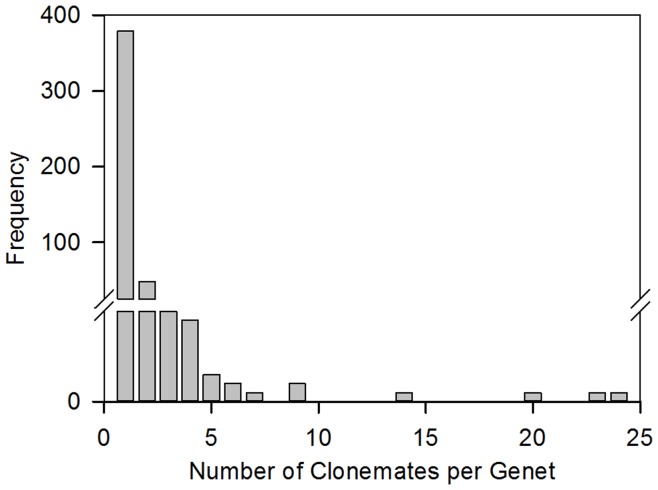
Frequency distribution of genet size for populations of *Montastraea annularis* across the Caribbean. In total n = 466 genets containing 698 clonemates were observed.

A total of 698 samples were successfully genotyped from 18 sites, identifying 466 multilocus genotypes (genets). Of these genets, 81% consisted of a single clonemate, 18% were represented by 2 to 14 colonies and the remaining 2% of genets were comprised of over 20 colonies (Fig. 2). The number of clonemates per genet differed among sites (Kruskal-Wallis H = 31.05, p = 0.020, df = 17), with Buoy 1 (Curaçao) having significantly more clonemates per genet compared to the other sites.

**Figure 3:**
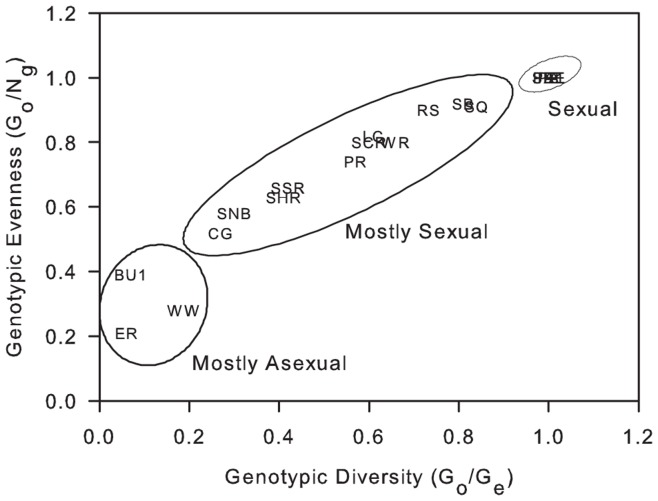
Clonal Structure of *Montastraea annularis* populations across the Caribbean. Based on the relationship between genotypic diversity and genotypic evenness, populations (n = 18) have been divided into 3 groups ranging from sexual to mostly asexual. Four of the five populations from Colombia are overlapping.

**Figure 4:**
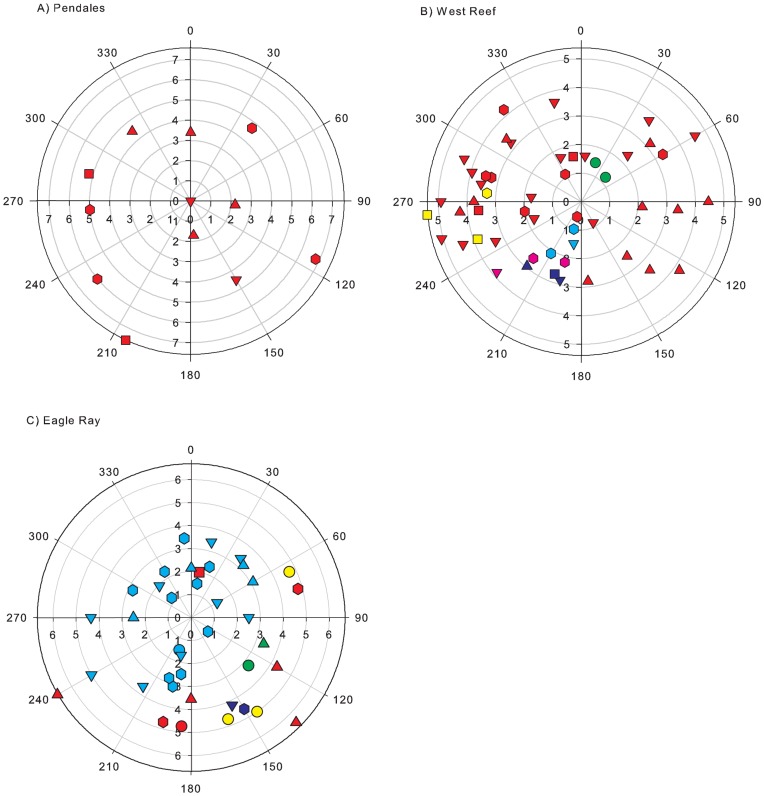
Polar plots of Montastraea annularis populations at three sites showing the distribution of colonies. a) Pendales, Cartagena; b) West Reef, Glovers Reef; and c) Eagle Ray, Caye Caulker. Each mark represents a colony. Genets represented by a single individual are indicated in red. Individuals of the same genet are indicated by the same colour. Size classes are denoted by shape (circle = 0–0.0300 cm^3^; square = 0.0301–0.1000 cm^3^; upward-triangle = 0.01001–0.2600 cm^3^; downward-triangle = 0.2601–0.8000 cm^3^; hexagon = 0.8001–17.500 cm^3^). Radial axis represents distance in m; angular axis represents angle in degrees. Number of colonies is 12, 51 and 39, respectively. All M. annularis colonies present within the circles were sampled.

Variation in clonal structure was represented by indices of genotypic diversity and evenness (Table 5). Both indices ranged from a value of almost 0 (where a few genets dominated) to 1 (where each genet was represented by a single colony) for all 18 sites sampled. The proportion of asexually produced colonies differed among the 18 sites (Kruskal-Wallis H = 32.24, p = 0.014). For example, Buoy 1 (Curaçao) and Eagle Ray (Caye Caulker) displayed a high proportion of asexual recruitment compared to Pendales (Cartagena), where every colony was unique. The average ratio of sexual to asexual reproduction (genotypic diversity) observed at the locations studied was moderately high at 0.59±0.32. The relationship between genotypic diversity and genotypic evenness was used to distinguish three groups of clonal structure within the 18 sites (Fig. 3). Four sites were shown to be sexual with an average genotypic diversity of 1.00±0 and an average genotypic evenness of 1.00±0, indicating that all the genets at the site were unique (Fig. 4a). Eleven sites were found to be mostly sexual with an average genotypic diversity of 0.57±0.19 and an average genotypic evenness of 0.75±0.14, indicating the occurrence of limited asexual reproduction but without any one genet dominating the site (Fig. 4b). Three sites were shown to be mostly asexual with an average genotypic diversity of 0.11±0.07 and an average genotypic evenness of 0.29±0.09, demonstrating that one or two large genets dominated the site (Fig. 4c).

**Table 5:** Genotypic diversity summary of *Montastraea annularis* colonies sampled from 8 regions of the Caribbean.

Region	Reef^a^	Area (m^2^)	Col Dens^b^	Genet Dens^c^	N^d^	N_g_ e	G_o_ f	G_o_/G_e_ g	G_o_/N_g_ h	Mean ColoniesPer Genet
Roatan	SB	78.5	0.61	0.47	42	37	33.92	0.81	0.92	1.14
	SQ	78.5	0.68	0.61	52	48	43.61	0.84	0.91	1.08
	WW	78.5	0.57	0.37	43	29	8.15	0.19	0.28	1.48
Caye Caulker	CG	126.7	0.35	0.18	44	23	11.95	0.27	0.52	1.91
	ER	141.0	0.28	0.09	39	12	2.53	0.06	0.21	3.25
Glovers Reef	LC	98.5	0.47	0.35	46	34	27.84	0.61	0.82	1.35
	WR	91.6	0.56	0.46	51	42	33.8	0.66	0.80	1.21
San Salvador	SHR	169.7	0.22	0.15	38	25	15.70	0.41	0.63	1.52
	SSR	149.6	0.33	0.21	50	32	21.19	0.42	0.66	1.56
New Providence	SCR	88.3	0.41	0.31	36	27	21.60	0.60	0.80	1.33
	PR	109.4	0.44	0.34	48	37	27.4	0.57	0.74	1.30
Curaçao	BU1	78.5	0.89	0.15	70	12	4.71	0.07	0.39	5.83
	SNB	78.5	0.76	0.40	60	31	17.82	0.30	0.58	1.94
San Bernardo	RS	237.8	0.05	0.04	11	9	8.067	0.73	0.90	1.22
	SBE				16	16	16	1.00	1.00	1.00
Cartagena	PE	176.7	0.07	0.07	12	12	12	1.00	1.00	1.00
	PA1				20	20	20.00	1.00	1.00	1.00
	PA				20	20	20.00	1.00	1.00	1.00
**Total**	18 reefs	1781.8			698	466				
**Mean**		118.8	0.45	0.28	38.78	25.89	19.24	0.59	0.73	1.67
**SD**		47.7	0.24	0.17	16.78	11.31	10.96	0.32	0.25	1.17

a Reef abbreviations provided in Table 1.

b Number of colonies m^−2.^

c Number of genets m^−2.^

d Number of colonies genotyped.

e Number of unique genotypes (genets).

f Observed genotypic diversity.

g Relative contribution of sexual and asexual reproduction.

h G_o_/N_g_, genotypic evenness.

### Colony Size

The size of colonies differed among the 18 sites (F = 22.37, p<0.001) and the 3 clonal groups (F = 69.21, p<0.001), with colonies in the sexual group being larger than the colonies in the mostly sexual and mostly asexual groups. In addition, clonemates were shown to be significantly smaller than non-clonemates across all 18 sites (F = 46.31, p<0.001).

**Figure 5:**
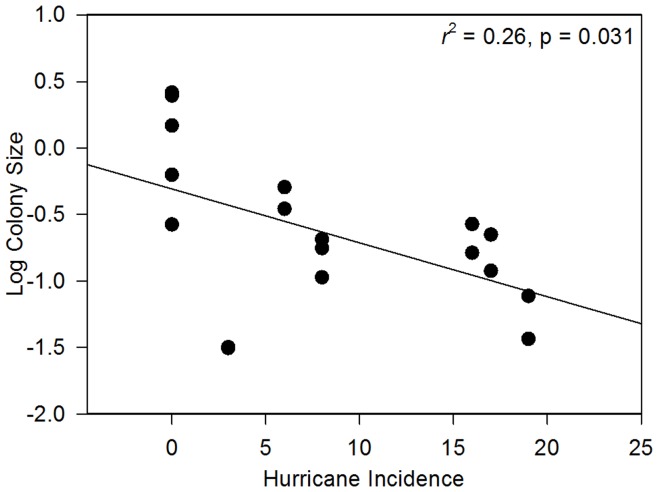
Relationship between colony size and hurricane incidence in *Montastraea annularis* populations across the Caribbean. n = 18 sites.

**Figure 6:**
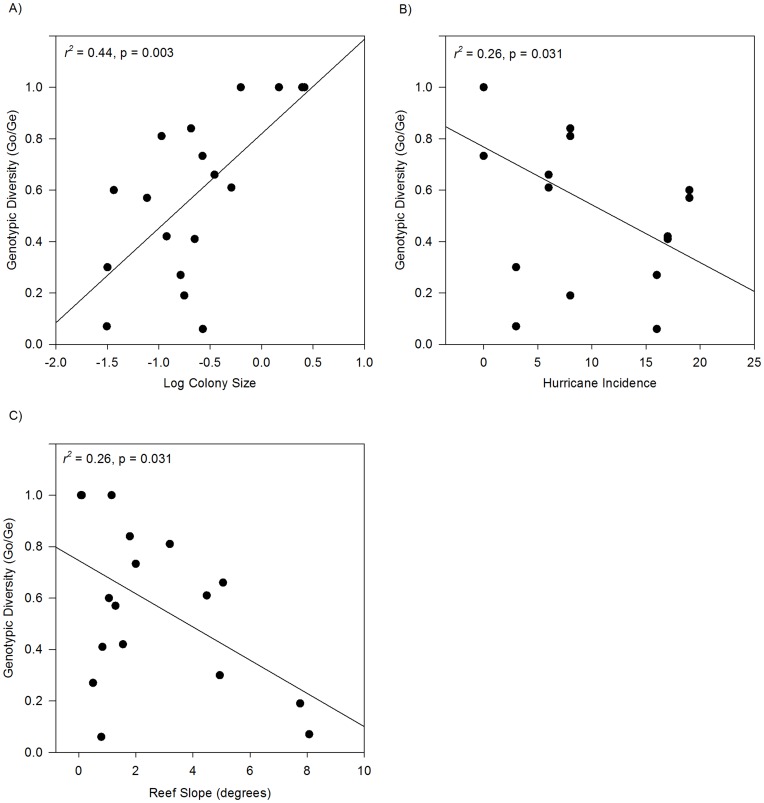
Relationship between genotypic diversity (G_o_/G_e_) and physical parameters in *Montastraea annularis* populations across the Caribbean. a) Colony Size; b) Hurricane Incidence; and c) Reef Slope. Higher genotypic diversity is associated with a) larger colony size; b) lower hurricane incidence; and c) gentler reef slope. n = 18 sites.

Interestingly, 26% of the variation in mean colony size among sites was explained by hurricane incidence at each site (β = −0.04, p = 0.031). Those sites experiencing a greater number of hurricanes were composed of smaller colonies than those sites experiencing less frequent hurricanes (Fig. 5). Furthermore, 44% of the variation observed in genotypic diversity among sites was explained by colony size, with larger colonies dominating sites with a higher genotypic diversity index (Fig. 6a).

### Spatial Distribution of Clonemates

The distance among clonemates ranged from a minimum of 0.05 m to a maximum of 13.2 m and differed significantly among sites (Moods χ^2^ = 193.73, p<0.001). Conservatively, we estimated that 90% of the clonal replication events involved breakage of the colony and dispersal of the fragments as distances among clonemates were greater than the average size of colonies at the 18 sites.

### Combination of Physical Parameters

A linear mixed effects model was created to test for competing hypotheses that might explain the observed pattern of genotypic diversity. The initial model included hurricane incidence, wave exposure, colony size, reef slope, and predicted input of *M. annularis* larvae for each site. Because hurricane incidence could arguably be non-independent at the scale of individual reef systems (region), which typically had two locations approximately 10 km apart, we added region as a random effect.

The only significant effects were hurricane incidence and reef slope (Table 6; Figs. 6b and 6c), both of which were highly significant (p<<0.05), explaining 64% of the variation in genotypic diversity among the 18 sites. Genotypic diversity was lower at sites located on steeper reef slopes and subject to higher hurricane incidence (Fig. 6b and 6c). Re-running the model without random effects gave a coefficient of determination, *r* ^2^, of 0.59 (p = 0.0004).

**Table 6:** Results of linear mixed effects models testing the effects of physical parameters on genotypic diversity (G_o_/G_e_) in 18 populations of *Montastraea annularis* across the Caribbean.

Model	Predictors	Model Coefficient (β)	T	p	df^a^	F	p^b^	*r^2^*
1	Constant	1.01	10.55	<0.001*	2, 15	13.16	<0.001*	0.64
	Hurricane Incidence	−0.03	−3.95	0.001*				
	Slope	−0.08	−3.95	0.001*				
2	Hurricane Incidence	−0.02	−2.37	0.031*	1, 16	5.62	0.031*	0.26
3	Slope	−0.07	−2.36	0.031*	1, 16	5.59	0.031*	0.26
4	Colony Size	0.37	3.55	0.003*	1, 16	12.59	0.003*	0.44

a Degrees of freedom.

b Associated probability.

* Significant at p<0.05.

## Discussion

The clonal structure of *M. annularis* was shown to vary considerably across the Caribbean, from genetically diverse populations in Colombia, where every colony was unique, to genetically depauperate populations in Belize and Curaçao, where a few genets dominated, adding support to previous findings of asexual reproduction in *M. annularis* [8], [51], [52]. We tested a number of hypotheses that could explain the observed pattern in genotypic diversity across the Caribbean, and from these hypotheses only hurricane incidence and reef slope were significant, supporting our hypothesis that major physical disturbances have a predictable positive impact on the incidence of asexual dispersal in this massive coral.

Those sites that experienced a greater number of hurricanes in the last 140 years were less genotypically diverse than those sites that experienced fewer hurricanes. Disturbance events have been documented to influence the proportions of sexual and asexual recruitment in terrestrial communities [10], [20], [70], [71]. Model simulations using both seedling and vegetative recruits predict that disturbance events that disrupt an area no greater than the dispersal distance of asexually generated recruits promote asexual recruitment [72]. Whereas, disturbance patches that extend beyond the distance of clone dispersal enhance sexual reproduction because asexual recruitment alone cannot efficiently exploit all the space created for settlement [72]. However, the results presented here show that increasing levels of disturbance, beyond the dispersal distance of clones, promote asexual reproduction within populations of *M. annularis*. Similar findings have been observed in populations of the trembling aspen, *Populus tremuloides*, where fires were demonstrated to increase levels of asexual reproduction [19]. Such deviations from the theoretical expectations of the life histories of clonal organisms may be partially explained by the varying effects of disturbance. Within populations of both *M. annularis* and *P. tremuloides* the disturbance events described are predicted to cause a direct proportion of asexual recruitment through physical fragmentation of colonies [51] and enhancement of sucker production [19], respectively, whereas disturbance events within the model simulation are programmed to result in 100% mortality of both sexual and asexual recruits. Within the 18 sites investigated here, at least 90% of multi-colony genets are highly likely to have been caused by physical breakage and dispersal, thereby reinforcing the idea that hurricanes promote asexual reproduction in *M. annularis*.

**Figure 7:**
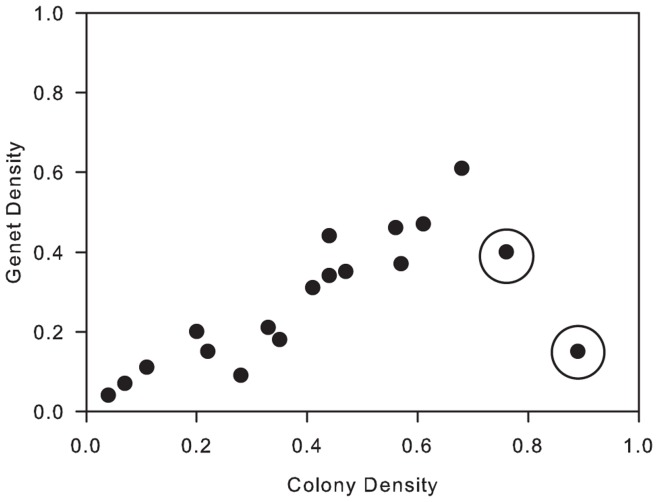
Relationship between colony density and genet density in *Montastraea annularis* populations across the Caribbean. Highlighted are the two sites at Curaçao. n = 18 sites.

It is important to note, however, that two sites sampled on the leeward coast of Curaçao had high levels of clonal structure despite being in an area with low hurricane incidence. One explanation for the occurrence of high levels of clonal structure at these two sites is that Hurricane Lenny passed within 200 miles of the island in 1999. The hurricane travelled on an unusual eastward path and 3–6 m high waves were reported to have pounded the leeward coast of Curaçao for 24 hours causing widespread damage to the reefs [73]. The effects of Lenny may be reflected in the clonal structure observed at the two sites in Curaçao. Furthermore, Curaçao receives relatively fewer sexually generated larvae as it has less potential upstream donor populations, and colonies grow so large, due to the absence of frequent hurricanes, that they fall apart and create clones (Vermeij pers. obs.). Both of these factors may have contributed to the unexpectedly high levels of clonal structure observed at the two sites in Curaçao. Here, genet density was higher than predicted based on colony density (Fig. 7). Removal of these two sites from the analysis strengthens our findings, with hurricane incidence alone then explaining 53% (p = 0.001) of the variation in genotypic diversity.

Previous studies of marine ecosystems have documented disturbance regimes as playing a role in determining the proportion of asexual recruitment within populations of clonal organisms [13], [16], [60], [74], [75]. However, they focus on species with a branching morphology. For example, Coffroth & Lasker [60] observed a correlation between genotypic diversity in populations of the gorgonian *Plexaura kuna* and current and wave action experienced by each site over an approximate 20 month period. Hunter [16] also documented a correlation between genotypic diversity and a combination of chronic and acute disturbance events in the branching coral *Porites compressa*. However, the lowest level of clonality was observed in the most severely or most recently disturbed populations. Interestingly, a stage-based simulation model identified both hurricane intensity and frequency as major factors in determining population structure in the branching coral *Acropora palmata* [76]. Although, genetic data were unable to confirm such patterns in the genotypic diversity of *A. palmata* populations in relation to hurricanes [15]. While we are not discounting acute disturbance as an important mechanism of dispersal in branching corals and gorgonians, it may be that the effects of such events are masked by more frequent chronic perturbations such as bad weather or winter storms, as discussed in Baums *et al* [15]. The fragile nature of branching morphologies makes them more susceptible to damage through wave action, and fragmentation is documented as a regular mechanism of colony dispersal [8], while in more massive colonies fragmentation may be restricted to periods of acute disturbance [77], [78]. In addition, particularly severe disturbance is likely to generate fragments from branching corals that are smaller than the critical size required for survival [79], whereas the robust columnar morphology of *M. annularis* colonies is more likely to give rise to larger fragments with a greater chance of survival.

The number of clonemates observed at a site was also related to the gradient of the reef slope. Sites on steeper reef slopes had a larger proportion of clonemates than those sites on more gentle slopes. Again, this is in contrast to findings in *A. palmata* where populations on gentler slopes were more clonal than populations on steeper slopes [15]. Baums *et al* [15] suggested that *A. palmata* fragments maybe lost from populations on steeper slopes at higher frequency. Size, shape and weight differences in fragments from columnar or massive colonies versus fragments from branching colonies may contribute to the observed contrasting patterns. In *M. annularis,* populations on steeper slopes may have higher asexual recruitment than those on more gentle slopes due to the moderating influence of slope on the impact of hurricane-induced waves. Waves generated by hurricanes have been observed to be larger and cause more damage to reefs on steeper slopes than on more gentle slopes [62], [63], [80]. Steeper reef slopes cause a sudden change in storm swells from deep water waves to shallow water waves, with little energy loss due to bottom friction, resulting in steep waves plunging vertically down on the reef causing extensive damage [63], [80]. On gentler sloping reefs, the change in water depth is less abrupt thereby increasing bottom friction, wave set-up is decreased and consequently the energy reaching the reef is reduced [81], [82]. In addition, an avalanche effect is created on steeper reefs whereby colonies near the top of the slope are uprooted or fragmented and cascade down the reef creating further damage, whereas on more gentle slopes many uprooted or fragmented colonies remain in place [83]. The avalanche effect may further increase the proportion of asexually generated offspring observed on reefs as fragments of colonies on steeper slopes are more inclined to move apart from the parent colony compared to fragments on a very gentle or flat reef. Here, fragments may be retained around the parent colony and later fuse to reform a single colony. Furthermore, steeper slopes may also promote survivorship of fragments by reducing sediment accumulation during storms.

The size distribution of colonies differed significantly among the 18 sites with clonemates being on average 70% smaller than non-clonemates. Furthermore, 26% of the variation in colony size was explained by hurricane incidence, with those sites that experienced more hurricanes having smaller colonies. Despite an extensive review of the literature we found no evidence to support a region wide variation in *M. annularis* growth rates consistent with latitude. The nature of clonemate formation, through fragmentation or partial mortality of the parent colony, is likely to result in colonies of a smaller size. Such differences in colony size were not observed in branching coral species, where clonemates and non-clonemates were documented as being similar in size [15], [16]. The faster growth rates of branching coral species may account for this lack of difference in size as fragments may quickly reach the average colony size within a population between successive disturbances. In contrast, *M. annularis* has a much slower growth rate, typically <10 mm yr^−1^ [44], thereby preventing fragments from rapidly increasing in size and thus maintaining a large difference in the size of clonemates and non-clonemates. Populations in which clonemates are significantly smaller than non-clonemates have been observed in the deep sea coral, *Lophelia pertusa* [18]. The smaller size of clonemates in *L. pertusa* was also linked to a reduction in fecundity [18]. However, the mean size of *M. annularis* clonemates in this study was much larger than the minimum size required for reproduction [84]. Nevertheless, corals have been reported to forgo sexual reproduction when subjected to stressful conditions such as bleaching or partial mortality [85], [86]. Thus, predicted increases in the frequency and severity of hurricanes [35], [36] could further reduce the ability of *M. annularis* populations to generate sexually produced larvae.

Remnant populations are described as those that persist through extended time periods, despite a negative population growth rate, due to long-lived life stages and life history characteristics buffering unfavourable environmental conditions and variability [87], [88]. Such life history characteristics include a high proportion of local asexual recruitment and high longevity of individuals within the populations [88]. Limited sexual recruitment within such populations may restrict recovery from disturbance and prevent colonisation of new habitats. Thus, current populations will decline at a rate determined by the longevity of existing individuals [87] in the absence of recruitment. Populations of *M. annularis* have declined in cover and abundance in recent decades with limited signs of recovery suggesting that growth rates [89], [90] and previously rare larval recruitment events are being increasingly inhibited by environmental changes on reefs [52], [91]. Size-based demographic models were used to predict trajectories of population growth for current *M. annularis* populations at St. John, US Virgin Islands [43]. The models predicted that current populations, with no further sexual recruitment, would be extirpated within 50 years. Moreover, populations may have declined to such an extent that even low sexual recruitment rates, typical of *M. annularis* populations, or the use of storage effects will be unable to sustain current population levels [43].

We demonstrate here that asexual reproduction occurs at varying frequency across the species-range and significantly contributes to the local abundance of a columnar, reef-building coral, providing further support to previous studies of asexual reproduction in M. annularis [8], [51], [52]. Large-scale disturbances combine with local habitat characteristics to shape the balance between sexual and asexual reproduction in populations of *M. annularis*. Although only 18 reefs were sampled in the current study, similar results would be expected across the remainder of the Caribbean. Reefs impacted by a greater number of hurricanes, such as those in the north and east of the basin, would be expected to have a greater proportion of asexual reproduction within the population. Reefs similar to those observed in Colombia, with low densities of large, solely sexual colonies, are likely to be rare.

Increasing levels of disturbance across the Caribbean may shift the balance further towards asexual reproduction resulting in stronger genet level selection. Long-term consequences may include increasingly isolated populations due to lower levels of dispersal of sexual propagules. A recent study of gene flow patterns in M. annularis across the Caribbean demonstrated that populations were genetically differentiated at a basin-wide scale, with discontinuities distinguishing populations in the eastern and western Caribbean and isolating the Bahamas [92]. Interestingly, distance was shown to be a poor predictor of gene flow in *M. annularis* suggesting that fine scale processes, such as larval life history traits, may significantly influence dispersal distances [92]. A shift towards a reliance on asexual reproduction may result in further reductions in gene flow among populations, with a subsequent loss of genetic diversity.

Asexual recruitment is clearly an important mechanism influencing the population structure of M. annularis. Our findings demonstrate that M. annularis can withstand acute disturbances, such as hurricanes, which would otherwise significantly reduce population density. Given the environmental and ecological changes occurring across the reefs of the Caribbean, this alternative method of recruitment may help buffer the adverse effects of utilising storage effects by generating offspring with significant advantages over sexual recruits. Recruits generated by fission or fragmentation (clonemates) are substantially larger than sexual recruits, and being raised above the substrate, may have a greater chance of survival because they are less subjected to sediment abrasion and algal competition [93]. Nevertheless, dependence on asexual methods of recruitment at the expense of sexual reproduction may have deleterious effects on the ability of M. annularis populations as a whole to adapt to climate change. Sexual populations can potentially evolve at a faster rate than asexual populations because beneficial mutations can be readily combined into an individual through sexual recombination [28]. Furthermore, sexual populations may be more tolerant of biotic stress through resistant genotypes already present in the genetically diverse population [94]. Future studies of the effects of climate-driven thermal and radiative stress on the coral/zooxanthellae holobiont [95] may need to consider a trend of reduced capacity for genetic diversification in some foundation coral species in the forthcoming decades.
